# Draft Genome Sequence of *Vibrio parahaemolyticus* Strain M1-1, Which Causes Acute Hepatopancreatic Necrosis Disease in Shrimp in Vietnam

**DOI:** 10.1128/genomeA.01468-17

**Published:** 2018-01-18

**Authors:** Ramya Kumar, Che-Chih Chang, Tze Hann Ng, Jiun-Yan Ding, Ta-Chien Tseng, Chu-Fang Lo, Han-Ching Wang

**Affiliations:** a Department of Biotechnology and Bioindustry Sciences, National Cheng Kung University, Tainan, Taiwan; b Center for Shrimp Disease Control and Genetic Improvement, National Cheng Kung University, Tainan, Taiwan

## Abstract

We report here the genome sequence of *Vibrio parahaemolyticus* strain M1-1, which causes a mild form of shrimp acute hepatopancreatic necrosis disease (AHPND). Compared to other virulent strains, the M1-1 genome appeared to express several additional genes, while some genes were missing. These instabilities may be related to the reduced virulence of M1-1.

## GENOME ANNOUNCEMENT

In the context of acute hepatopancreatic necrosis disease (AHPND), *Vibrio parahaemolyticus* strains can be described as either virulent or nonvirulent. The ability of a given strain to cause AHPND depends on the presence of the virulence plasmid pVA1, which harbors the binary toxins PirA^*Vp*^ and PirB^*Vp*^ (1). We previously reported that for three strains of *V. parahaemolyticus* isolated from Thailand, two strains, 3HP and 5HP, caused AHPND, while the third strain, S02, did not (1). Similarly, for two other strains collected from AHPND-infected pond water in Vietnam, M1-1 was found to be an AHPND-causing strain, while M2-36 was a non-AHPND strain with a deletion of the *pirAB* gene fragment on the pVA1 plasmid (1). However, although M1-1 harbored the pVA1 plasmid and expressed Pir toxins, it showed only mild virulence compared to the 3HP and 5HP strains. Evidence of this relatively mild virulence includes the fact that shrimp challenged with M1-1 had a lower mortality rate than those challenged with 3HP or 5HP (2). Intriguingly, we noted that when M1-1 was maintained as a plate or broth culture for a prolonged period (2 to 3 weeks), the *pirAB* gene fragment was lost, even though the pVA1 plasmid was still present. In contrast, the loss of the *pirAB* gene fragment has not been observed in the virulent strains 3HP and 5HP. To further investigate the possible reasons for this loss of the *pirAB* gene fragment from the pVA1 plasmid, we sequenced the M1-1 strain and compared its genome with those of the 3HP, 5HP, and S02 strains reported previously (3).

Bacterial genomic DNA was extracted using a FavorPrep tissue genomic DNA extraction kit (Favorgen, Taiwan). Genomic libraries were prepared using a NEBNext ultra DNA library preparation kit (New England Biolabs) and sequenced using the Illumina MiSeq platform. The sequence data were assembled into 307 contigs using the Velvet *de novo* assembler (4), and the contigs were annotated with the Rapid Annotations using Subsystems Technology (RAST) server (5) by comparing the M1-1 strain with *V. parahaemolyticus* strains 3HP, 5HP, and S02. The contigs of M1-1, 3HP, and 5HP were then compared against the reference genome of the AHPND-causing strain *V. parahaemolyticus* RMID 2210633. A high protein sequence identity of 99% was seen between the virulent strains of *V. parahaemolyticus*. However, there were also several important differences between 3HP, 5HP, and M1-1.

Specifically, we found that the M1-1 genome had several additional genes that were not present in either 3HP or 5HP. The functional categories of these genes included coding for DNA metabolism, the phage major capsid protein, the phage DNA replication protein, and stress response. We also found several other proteins that were present in 3HP and 5HP but absent in M1-1. These nine proteins include transketolase and endoglucanase proteins involved in carbohydrate metabolism, a ribosome modulation factor regulating sporulation and dormancy, conjugative transfer proteins TrbE, TrbG, TrbI, and TrbJ that help in membrane transport, a nitrite reductase complex (NrfF) facilitating nitrogen metabolism, and an electron transport complex protein (RnfC) involved in respiration. We anticipate that further investigation of these findings will lead to a better understanding of both M1-1’s loss of virulence and the molecular mechanisms that underlie the pathogenicity of AHPND.

### Accession number(s).

This whole-genome shotgun project has been deposited at DDBJ/ENA/GenBank under the accession number PDDQ00000000. The version described in this paper is the first version, PDDQ01000000.
